# Novel lncRNAs Co-Expression Networks Identifies LINC00504 with Oncogenic Role in Luminal A Breast Cancer Cells

**DOI:** 10.3390/ijms22052420

**Published:** 2021-02-28

**Authors:** Carolina Mathias, Clarice S. Groeneveld, Sheyla Trefflich, Erika P. Zambalde, Rubens S. Lima, Cícero A. Urban, Karin B. Prado, Enilze M. S. F. Ribeiro, Mauro A. A. Castro, Daniela F. Gradia, Jaqueline C. de Oliveira

**Affiliations:** 1 Post-Graduation Program in Genetics, Department of Genetics, Federal University of Parana, Curitiba 81530-900, PR, Brazil; carol.mathias1@hotmail.com (C.M.); erikazambaldi@gmail.com (E.P.Z.); kbraun@ufpr.br (K.B.P.); enilzeribeiro@gmail.com (E.M.S.F.R.); danielagradia@gmail.com (D.F.G.); 2 Cartes d’Identité des Tumeurs Program, Ligue Nationale Contre le Cancer, 75013 Paris, France; clari.groeneveld@gmail.com; 3 Oncologie Moleculaire, Institut Curie, CNRS, UMR144, Equipe Labellisée Ligue Contre le Cancer, 75005 Paris, France; 4 Bioinformatics and Systems Biology Laboratory, Polytechnic Center, Federal University of Parana (UFPR), Curitiba 81520-260, PR, Brazil; sheylatrefflich@gmail.com (S.T.); mauro.a.castro@gmail.com (M.A.A.C.); 5 Breast Disease Center, Hospital Nossa Senhora das Graças, Curitiba 80810040, PR, Brazil; rsilveiralima@uol.com.br (R.S.L.); cicerourban@hotmail.com (C.A.U.)

**Keywords:** LINC00504, breast cancer, co-expression, lncRNA, luminal A

## Abstract

Long non-coding RNAs (lncRNAs) are functional transcripts with more than 200 nucleotides. These molecules exhibit great regulatory capacity and may act at different levels of gene expression regulation. Despite this regulatory versatility, the biology of these molecules is still poorly understood. Computational approaches are being increasingly used to elucidate biological mechanisms in which these lncRNAs may be involved. Co-expression networks can serve as great allies in elucidating the possible regulatory contexts in which these molecules are involved. Herein, we propose the use of the pipeline deposited in the RTN package to build lncRNAs co-expression networks using TCGA breast cancer (BC) cohort data. Worldwide, BC is the most common cancer in women and has great molecular heterogeneity. We identified an enriched co-expression network for the validation of relevant cell processes in the context of BC, including LINC00504. This lncRNA has increased expression in luminal subtype A samples, and is associated with prognosis in basal-like subtype. Silencing this lncRNA in luminal A cell lines resulted in decreased cell viability and colony formation. These results highlight the relevance of the proposed method for the identification of lncRNAs in specific biological contexts.

## 1. Introduction

Non-coding RNAs are a big class of transcripts that can be classified according to their size, comprising small RNAs <200 nucleotides (nt) and long non-coding RNAs (lncRNA) >200 nt [1]. LncRNA molecules are usually transcribed by RNA polymerase II, capped, and polyadenylated with some being also spliced. LncRNAs present high tissue specificity and great regulatory versatility, acting at different levels of gene expression regulation [2,3]. LncRNAs have already been analyzed in several human diseases, including cancer, with varying regulatory activity as either oncogenic or tumor suppressor, whose activity can modulate all hallmarks of cancer [4]. For example, the lncRNA HOTAIR can promote tumor growth and metastasis in several cancer types, such as breast, hepatocellular, lung and gastric cancer. [5].One lncRNA known to act as a tumor suppressor, regulating p53, is the Maternally expressed gene 3 (MEG3). Several studies have shown down-regulation of this lncRNA in human cancers, such as lung, breast, gastric and colorectal [6].

LncRNAs display a highly tissue-specific expression, which can render them potential candidates for cancer diagnosis. Their expression also correlates with overall survival (OS), metastasis, tumor stage or grade, highlighting theirpotential use as prognostic markers [7].

Nevertheless, only a small portion of lncRNAs have been functionally characterized, and the function of most of them remains elusive. To better understand these molecules’ biology, various computational methods and tools for identifying, annotating, and performing functional prediction for long non-coding RNAs are being used extensively [8].

One of the most used approaches towards understanding the function of lncRNAs is based on co-expression patterns shared with protein-coding counterparts [9,10]. Varied methodologies have already been used to predict these co-expression networks; however, caution should be exercised when analyzing large patient cohorts’ data.

In this study, we propose to use the pipeline available from the Bioconductor/R package RTN. This method is tuned to deal with large gene expression datasets to build transcriptional regulatory units. It was previously used to compute networks regulated by transcriptional factors [11,12] and uses mutual information (MI) metric based on a gene’s expression varying across a cohort. In the present work, we used this approach to construct regulatory networks focused on units of co-expression networks due to the fact of its great statistical rigor for its analysis.

Breast cancer (BC) is a critical health problem worldwide. GLOBOCAN 2018 estimated the diagnosis of 2,088,849 new cases worldwide [13]. BC is a heterogeneous disease characterized by several pathological features, divergent treatment responses, and substantial differences in long-term patient survival [14]. According to gene expression profiles, BC may be classified mainly into four molecular subtypes, luminal A and B, HER2-enriched and basal-like. Luminal A and B are both sensitive to hormone therapy, although luminal A patients have often low-grade tumors and good prognosis while luminal B is recognized as having a higher proliferation rate. HER2-enriched and basal-like types are widely considered to have poorer survival and tumors with higher grade [15]. In relation to therapeutic conduct, positive estrogen subtypes benefit from treatment by Tamoxifen and HER2-enriched patients can benefit from monoclonal antibody therapy. Patients with basal-like tumors have poorer survival [16,17], and remain the most challenging group to treat, but new targeted therapies are becoming available, as, for example, PARP inhibitors in *BRCA* mutated patients. Here, we used lncRNA co-expression networks to identify important lncRNAs in BC by focusing on differences between molecular subtypes.

The results obtained through the use of the proposed methodology highlight the potential biological relevance of lncRNAs in BC. Therefore, this methodology could be used for screening of functional lncRNAs in different physiological and pathological contexts.

## 2. Results

### 2.1. LncRNAs Co-Expression Networks Activity Are Different among Breast Cancer Molecular Subtypes

To build the co-expression networks, we used data from 12,000 lncRNAs identified in the TCGA cohort. Since lncRNAs intrinsically have low expression, we selected only lncRNAs expressed in at least 90% of the tumor samples. This filter resulted in 3680 lncRNAs, that were then used to build the co-expression networks, in addition to mRNA expression data. In this way, co-expression networks could jointly present lncRNAs and mRNAs in their composition.

**Figure 1:**
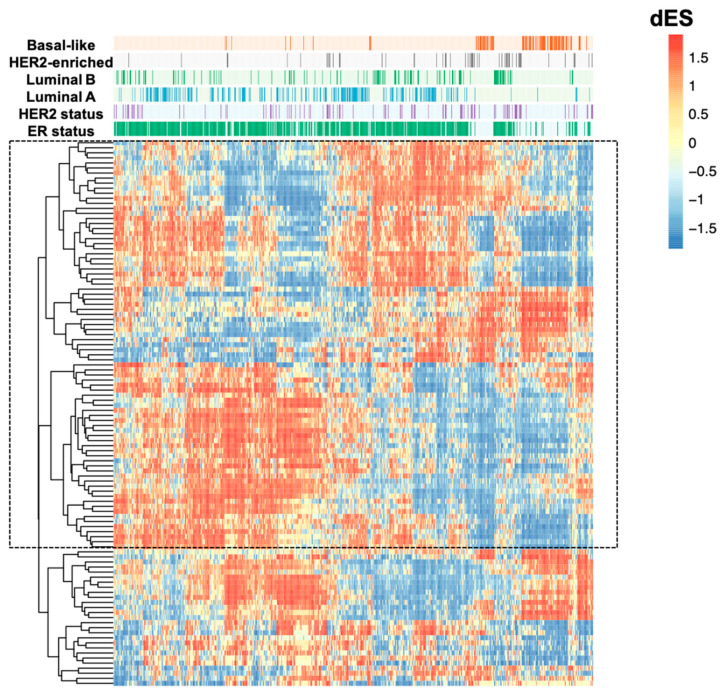
Heatmap representation of the 84 lncRNA co-expression networks activity in breast cancer molecular subtypes. According to this metric, positive dES (in red) are activated; and negative dES (in blue) are inactivated. In the highlighted rectangle are the more contrasting between luminal A and basal-like subtypes. The samples are organized in the heatmap columns and categorized according to the annotation bar at the top of the graph. The TCGA BRCA cohort samples were classified according to their molecular classification and estrogen receptor and HER2 status. LncRNAs in their co-expression networks (*n* = 84) are clustered in the rows and can be found in Supplementary Table S2.

Next, we looked for networks that had characteristics intrinsic to their statistical construction, which could reflect greater biological relevance in the analyzed context. The networks were first filtered according to their size, those containing less than 15 genes were exceeded, and then, according to Differential Enrichment Score (dES) −0.5 < dES > 0.5 values, obtaining 84 lncRNA co-expression networks (Supplementary Table S2). As represented in Figure 1, many differences can be identified, mainly between the luminal A and basal-like subtypes, which make up the most clinically contrasting subtypes. The most significant contrasts between these subtypes are emphasised in the figure. To identify lncRNAs co-expression networks significantly different between these two subtypes, we used Kruskal-Wallis tests to statistically determine the co-expression networks differentially expressed in these two subtypes using the dES metrics. At this point, the data were filtered according to the higher numeric differences between the luminal A and basal-like subtypes due to the great clinical contrast of these subtypes. In this last step, we obtained a list of 40 lncRNA co-expression networks (Supplementary Table S3).

### 2.2. LncRNAs Co-Expression Networks Are Enriched for Cancer-Related Function

The 40 co-expression networks filtered, according to criteria described in the previous results section, were analyzed for biological process enrichment. This enrichment analysis was performed using the molecular signature data “Hallmarks” deposited in the MSigDB database.

**Figure 2:**
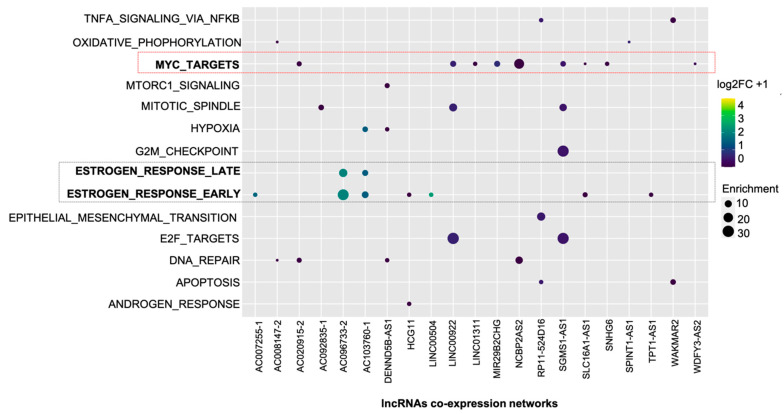
Enrichment map of the identified co-expression networks. In rows are the 14 identified molecular signatures, and in the columns, the networks of co-expression of 21 lncRNAs. Dot size refers to the number of genes identified in each cancer-related process. Blank spaces mean that there was no statistically significant enrichment. Dot color refers to the difference (logFC + 1) of the lncRNAs used as a reference for building co-expression networks. The comparison was made between luminal A and basal-like, with dots with colors closer to yellow more expressed in luminal subtype A. Colors closer to purple indicate down-regulation in luminal A. In this enrichment map, the pathway named “MYC_TARGETS” (red rectangle) and “ESTROGEN_RESPONSE_EARLY” (black rectangle) stands out as the most frequently identified.

Among these co-expression networks, 21 of them were statistically enriched (*p* < 0.01) for some cancer-related function (Figure 2).

Among the 50 datasets available in “Hallmarks” collection, 14 were identified in the analyzed co-expression networks. The most frequently identified were “MYC_TARGETS” (*n* = 9) and ESTROGEN_RESPONSE_EARLY (*n* = 7). The results obtained highlight the consistency of the RTN pipeline’s data since the estrogen and *MYC* oncogene signaling pathways are known to be relevant in the process of mammary carcinogenesis.

To biologically validate the relevance of one of the lncRNAs used in constructing the co-expression networks, we chose the one that presented the wider dES range comparing the luminal A and basal-like subtypes. This lncRNA is named LINC00504 (ENSG00000248360), and according to Figure 2, it has a significative value for “ESTROGEN_RESPONSE_EARLY” enrichment.

### 2.3. LINC00504 Co-Expression Network in Breast Cancer Luminal-A and Basal-Like Subtypes

**Figure 3:**
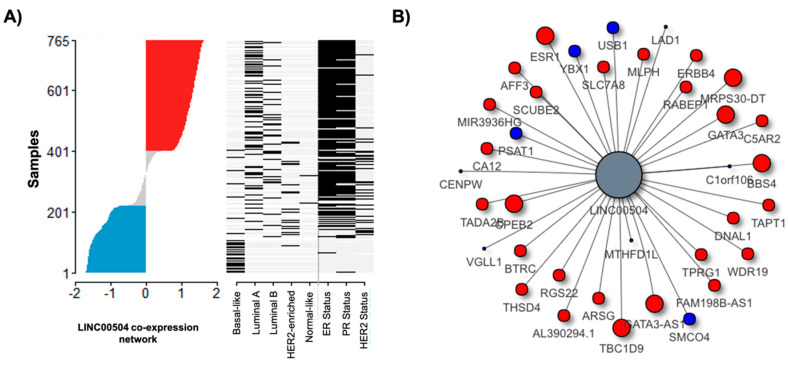
Network activity and LINC00504 co-expression network representation. (**A**) Ranked dES plot for the BC cohort and status of key attributes plot. Each dash in the status attributes plot represents a patient. The cohort of patients was extracted according to their molecular classification and immunohistochemical parameters (estrogen and progesterone receptors and HER2 status). Positive dES, indicated by the color red, represents the up regulation of this network along the samples, whereas negative dES is indicated by the color blue. In this case, positive regulon activity is located in Luminal A patients; (**B**) Network representation of LINC00504 zoomed in on genes with higher mutual information values. Red circles refer to genes with positive correlation and blue to negative ones. Dot size refers to the mutual information value, where big dots indicates higher mutual information.

We organized LINC00504 co-expression network data in a ranked dES plot for the BC cohort (Figure 3A). This graph highlights the activation of these networks in luminal A patients and positive for estrogen and progesterone receptors. Figure 3A also shows that basal-like patients are grouped in negative network activity (represented in blue). Genes already positively related to luminal subtype A, such as transcription factors (TFs) *ESR1*, *GATA3*, and *FOXA1*, can be recognized. According to our data, these TFs have positive mutual information values with LINC00504, meaning the concordant expression variation among these molecules (Figure 3B). The graphical representation of the co-expression network was limited to genes with high mutual information values for better visualization.

### 2.4. LINC00504 Is Up-Regulated in Luminal A and Is Related to Better Prognosis in Basal-Like Subtype

**Figure 4:**
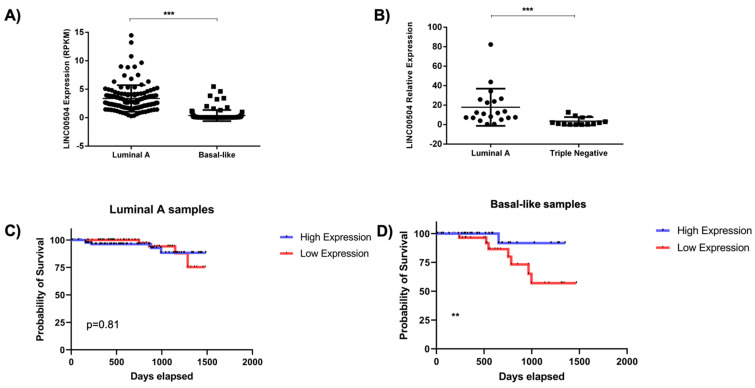
LINC00504 expression and its relationship with patients’ survival. (**A**) LINC00504 expression (RPKM) in TCGA BRCA cohort. In total, 231 luminal A and 98 basal-like samples were used, and LINC00504 is up-regulated in luminal A patients; (**B**) LINC00504 relative expression in a Brazilian’s patients’ cohort. In this cohort, 30 luminal A and 25 triple-negative samples were used, and we observed the same pattern of up-regulation in luminal A samples; (**C**,**D**) Survival analysis considering median LINC00504 expression value as cut-off in Luminal A samples and Basal-like samples, respectively. Red and blue lines refer to patients with low and high expression, respectively. ** *p* < 0.05 and *** *p* < 0.01.

After identifying LINC00504 as a potential relevant lncRNA in mammary carcinogenesis, we focused on the functional characterization of the LINC00504. Since its co-expression network has been identified with the significant difference between the luminal A and basal-like subtypes, we first evaluated its expression at the TCGA cohort. We found a significant difference in LINC00504 expression between Luminal A (*n* = 231) and basal-like (*n* = 98) patients (*p*-value < 0.05). LINC00504 was up-regulated in luminal A samples (*p* < 0.01) at the TCGA cohort (Figure 4A).

The same comparison between subtypes was made in an independent Brazilian cohort (*n* = 55) by RT-qPCR. In this case, the samples were classified as triple-negative since the molecular classification information is not available in the patients’ clinical reports. In these samples, we also found LINC00504 up-regulation of in luminal A samples (*p* < 0.01) (Figure 4B). 

The next step was to analyze this lncRNA expression’s clinical impact on the patient’s survival inside each subtype using TCGA cohort follow-up data. The median and quartile values (P25 and P75), were used as cut-offs for the groups, with no difference in survival in luminal A patients (Figure 4C). Notably, basal-like patients with higher median expression of LINC00504 had a higher survival probability (*p* < 0.05). Therefore, in the basal-like subtype, the high expression of LINC00504 is related to the better prognosis (Figure 4D).

### 2.5. LINC00504 Is Related to Colony Formation and Cell Viability in Luminal A Cell Lines

**Figure 5:**
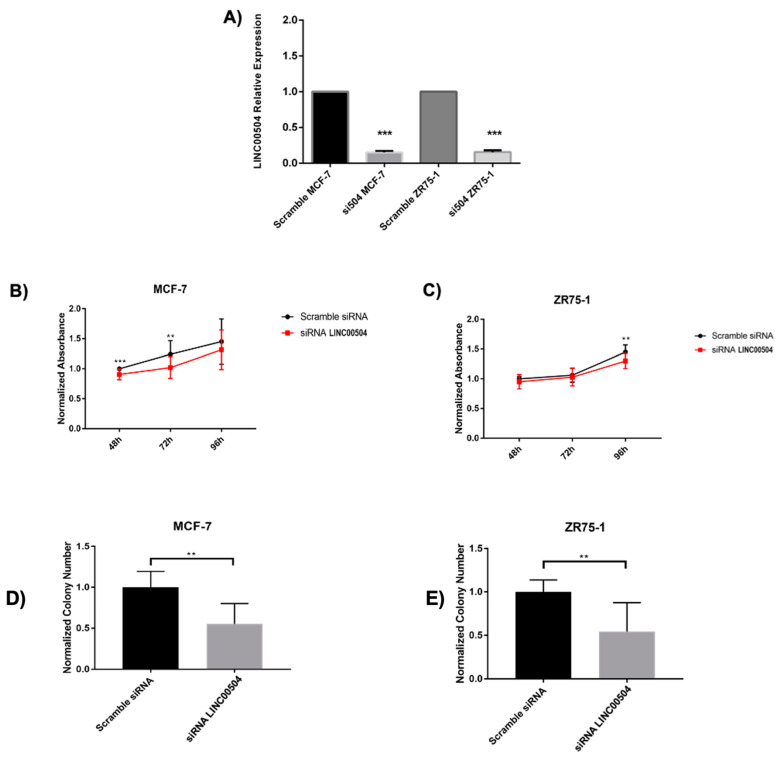
Functional validations of LINC00504 in luminal A cell lines. (**A**) LINC00504 silencing efficiency 48 h after transfection; (**B**) Cell viability in MCF-7 cell line; (**C**) Cell viability in ZR75-1 cell line; (**D**) Colony formation in MCF-7 and (**E**) Colony formation in ZR75-1 cell line; *** *p* < 0.01, ** *p* < 0.05.

To verify the potential biological relevance of LINC00504 in luminal A subtype, we silenced its expression by using the RNA interference technique in two different cell lines, MCF-7 and ZR75-1. These cell lines were chosen because they have a high expression of LINC00504 and are therefore suitable for performing a silencing test (Figure S1). The most efficient result (90% of silencing) was achieved after 48 h after transfection (Figure 5A).

The silencing of LINC00504 decreased the cell viability as measured by Resazurin assay, of the MCF-7 48 h (*p* < 0.01) and 72 h (*p* < 0.05) (Figure 5B) after treatment. For the ZR75-1 cell line, we observed this phenotype 96 h (*p* < 0.05) after treatment (Figure 5C). The number of colonies was significantly reduced for both cell lines after the lncRNA silencing (Figure 5D,E and Figure S2). Apoptosis investigation assays were also carried out on the same strains after the silencing of lncRNA. No statistically significant value was found in this assay.

### 2.6. LINC00504 Co-Expression Network Has Transcriptional Factors Related to Its Regulation

As previously shown in Figure 3B, the LINC00504 co-expression network contains TFs already related to the luminal A subtype, such as *GATA3*, *FOXA1* and *ESR1*. These TFs were identified in this network, presenting positive mutual information values to LINC00504, thus positively related. With this, it can be assumed that the variation in these RNAs’ expressions occur in the same direction, increasing or decreasing together. From the data of mutual information of all the elements identified in this co-expression network, it can also be verified that these genes were the ones that presented higher values of mutual information (Table 1).

**Figure 6:**
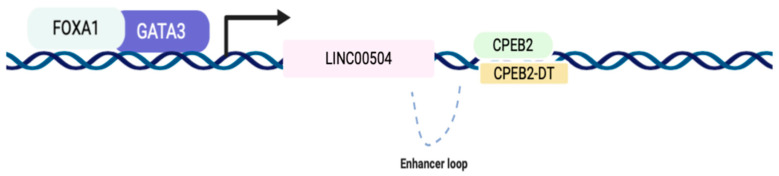
Scheme of transcriptional regulation mediated by GATA3 and FOXA1 in the promoter region of LINC00504. From public data from ChIP-Seq, it was identified that these transcription factors (TF) bind to the promoter region of LINC00504, possibly activating its transcription. These TFs were also identified in the LINC00504 co-expression network.

When comparing these RNAs’ expression values, considering luminal A x basal-like, it is observed that *ESR1*, *FOXA1,* and *GATA3* are up-regulated in luminal subtype A, justifying the positive values of mutual information, since LINC00504 is also up-regulated in luminal A subtype. Based on ChIP-Seq data, made available by the ENCODE project [18,19], it is experimentally evidenced that the GATA3 and FOXA1 bind in the promoter region of LINC00504, possibly regulating its transcription (Figure 6).

Another mRNA that stood out for having positive value for mutual information encodes for the Cytoplasmic Polyadenylation Element Binding Protein 2 (*CPEB2*). This protein is required for cell cycle progression, specifically for the transition from metaphase to anaphase [20]. *CPEB2* was shown to promote differentiation and inhibit the epithelial-to-mesenchymal transition in mammary epithelial cells [21]. *CPEB2* elongates the poly(A) tail length of CPE-containing mRNAs, regulating post-transcriptionally mRNAs downstream of steroid hormone signaling, emphasizing the importance of this regulatory molecule in the development of luminal A subtype. Published data from *CPEB2* RNA immunoprecipitation [22] identified 169 mRNAs co-immunoprecipitated with *CPEB2* in mammary epithelial cells. We identified 41 of these molecules, also present in LINC00504 co-expression network (Supplementary Table S4). Among these mRNAs, *CCND1*, *IGFBP4*, *TIPARP* and *UGCG* belong to the genetic signature “ESTROGEN_RESPONSE_EARLY”, identified in the LINC00504 co-expression network enrichment, emphasizing the relevance of this network to the luminal A phenotype.

Through a detailed analysis of the genomic region where LINC00504 is located, we found that the *CPEB2* is located downstream, on the opposite strand. This genomic region is also characterized by the abundance of H3K27Ac epigenetic marks, which are found in regulatory elements, including active enhancers [23]. In fact, when analyzing this region using data deposited on the Genome Browser platform (UCSC), we found a characteristic enhancer loop in the region that comprises these genes, illustrated in Figure 6 It can then be suggested that LINC00504 acts as a trans-enhancer by activating *CPEB2* transcription. This finding requires experimental validation and may represent an important regulatory dependency found in the luminal subtype A.

These results emphasize the robustness of the method used to identify the co-expression networks and identify important lncRNAs. This is the first study to describe the relevance of LINC00504 to breast cancer, and due to the connections established from its network, we show this lncRNA can be important for the luminal A subtype.

## 3. Discussion

Breast cancer is a heterogeneous disease, encompassing numerous subcategories with differing cellular compositions, molecular alterations, and clinical follow-ups. Due to this heterogeneity, breast cancer classification is an important aspect of therapeutic decision-making [24]. BC can be classified according to histological, immunohistochemical, and molecular profiles. The current BC molecular classifications measure mRNAs expression levels and do not consider non-coding RNAs. However, lncRNAs are gaining prominence in studying molecular profiles of cancer [25,26] since they present remarkable tissue-specific expression [27].

Many computational methods have been developed to understand and characterize the biology of lncRNAs in physiological and pathological conditions [28,29]. In this work, we propose to use the pipeline contained in the RTN package to search for lncRNAs co-expression networks that provide evidence of their biological relevance in BC. This methodology brings some advantages to others developed with the same objective: Its development was focused on the study of large patient cohorts, and allows the user to set the stringency of the analysis in a stepwise process, including a bootstrap routine designed to remove unstable associations.

We used this strategy to search for lncRNAs co-expression networks with relevance in breast cancer, mainly comparing the luminal A (better prognosis) and basal-like (poor prognosis) subtypes. After using filters, due to the expressive number of results obtained, we call attention to a specific lncRNA (LINC00504) that has not yet been studied in breast cancer and presented exciting results in network analysis.

LINC00504 co-expression network was identified as one with the most significant differences in Differential Enrichment Score (dES) between luminal A and basal-like subtypes. A large positive dES indicates an induced (activated) regulon, while a large negative dES indicates a repressed regulon [30,31]. 

We identified that LINC00504 is significantly increased in luminal A subtype compared to basal-like. Besides, there was an impact of LINC00504 expression with better prognosis in basal-like patients. Functional studies carried out on luminal A cell lines have shown that LINC00504 down-regulation decreases cell viability and colony formation (Figure 5).

Similar to what we observed in breast cancer cell lines, the silencing of LINC00504 reduced cell viability, colony formation and cell migration in colon cancer cell lines [32]. In the same study, LINC00504 silencing also decreased xenograft tumor volume, suggesting that LINC00504 promoted colon cancer development both in vitro and in vivo. In lung cancer cells, LINC00504 expression was higher than in normal cell lines, and its silencing significantly inhibited cell proliferation, colony formation, invasion, and migration and promoted cell apoptosis. Based on these results, LINC00504 was suggested to play an oncogenic role in lung cancer [33]. Another study in lung cancer demonstrated that LINC00504 up-regulation is associated with aggressive progression and poor prognosis in non-small cell lung cancer [34]. The decrease in cell viability and formation of colonies after silencing the LINC00504 was also identified in a study using ovarian cancer cells, indicating that LINC00504 may play an oncogenic role in ovarian cancer cells [35]. These results, in agreement with the results presented in the present study, highlight the relevance of LINC00504 in breast cancer luminal subtype A, suggesting its role as an oncogene in this subtype.

Exploring the data from the co-expression network identified for LINC00504, we highlight here mRNAs that presented the highest positive values of mutual information: *CPEB2*, *ESR1*, *FOXA1* and *GATA3*. 

The TFs *FOXA1* and *GATA3*, have been experimentally validated to bind LINC00504 promoter’s region, potentially inducing its transcription, according to ENCONDE project public data. The regulation of *ESR1* and LINC00504 has not yet been experimentally validated; however, we know that GATA3 and FOXA1 mediate ESR1 binding to the *cis*-regulatory elements that drive transcription of the *ESR1* target genes. This GATA3 mediation is one of the central components of the ESR1 complex that determines the binding potential and transcriptional targets in breast cancer cells [36]. As suggested by our analyses, these RNAs present positive mutual information, thus varying in the same direction. Therefore, the understanding of the regulatory network of LINC00504 that links these elements together has great biological relevance to the study of luminal A subtype.

According to our analyses, *CPEB2* was the mRNA that presented the highest value of mutual information with LINC00504 (Table 1). CPEB2 regulates the poly(A) tail length of CPE-containing mRNAs, contributing to mammary gland development and luminal breast carcinogenesis by regulating the translation of mRNAs downstream of steroid hormone signaling [22]. Pascual et al. 2020 [22], using METABRIC and TCGA breast cohorts, verified that *CPEB2* is associated with *ESR1* levels, and high levels of *CPEB2* were associated with worse survival compared to samples with the lowest expression in luminal A patients. On the other hand, ER^−^ tumors (such as basal-like) do not seem to require *CPEB2*; low levels of *CPEB2* result in reduced survival. 

In this same work [22], the authors induced the silencing of *CPEB2* (through the shRNA method) in ZR75-1 cell line, and this depletion significantly decreased cell proliferation in vitro but did not increase apoptosis. Our LINC00504 silencing experiments had similar results to those found in the work of Pascual et al. 2020. This may suggest a mechanism of *CPEB2* and LINC00504 regulation. 

*CPEB2* is one of the top six genes, together with *ESR1*, with the strongest correlation with ER^+^ breast cancer prognosis [36]. Although the cell-of-origin for luminal tumors has not yet been unambiguously identified, these tumors appear to arise from a population of ductal progenitor cells, which have clonogenic capacity and express high levels of markers of mature luminal cells, such as ER, PR, *GATA3*, and *FOXA1* [37,38].

The lncRNA LINC00504 seems to be located in an important regulatory region, which shows high H3K27Ac markers, usually founded in regulatory elements [18]. Through a detailed analysis of this genomic region, it is possible to identify the formation of an enhancer loop housing the LINC00504, CPEB2 and CPEB2-DT genes (Figure 6). This regulatory loop has not yet been experimentally explored and may bring new key regulatory mechanisms in developing luminal subtype.

In summary, the results obtained from the construction of the co-expression network proved to be robust, after considering the 40 networks resulting from the applied filters, by the identification of pathways already related to enriched breast cancer. In addition, we highlight the results found for the LINC00504 co-expression network. In this network, we identified RNAs previously related to each other, and with the luminal subtype A, according to data available in the literature. In addition, functional studies in cells of the luminal subtype A with this lncRNA, have shown to be in agreement with those previously performed in other tumor types. Therefore, we suggest that this pipeline for the construction of regulatory networks can be used in future studies, considering large cohorts of patients, to elucidate relevant biological aspects in the development and progression of the disease.

## 4. Materials and Methods

### 4.1. TCGA Data Extraction

The expression data of the lncRNAs were extracted from the TANRIC platform (https://ibl.mdanderson.org/tanric/_design/basic/main.html (accessed on 1 January 2021)). In this database, the TCGA cohort expression data (TCGA, Nature 2012), the BAM files used RPKM to quantify the expression levels of lncRNAs. The mRNA data was extracted from the same TCGA project using the Firebrowse platform (http://firebrowse.org (accessed on 1 January 2021)). The clinical data of the patients in this study were extracted using the cBioPortal platform (https://www.cbioportal.org (accessed on 1 January 2021)).

### 4.2. RTN Pipeline Implementation

The RTN package is designed for the reconstruction of the transcriptional regulatory network (TRNs) and analysis of regulons using mutual information (MI) [11]. It is implemented by S4 classes in R and extends several methods previously validated for assessing regulons, e.g., MRA [39], GSEA [40], and EVSE [12]. 

An expression matrix, grouping data from lncRNAs and mRNAs, was organized using the samples that presented the two available data. To select the lncRNAs that would be considered to construct the co-expression networks, we applied a filter under the total number of lncRNAs. Only lncRNAs with detectable expression in at least 90% of the tumor samples used were selected. As a result, 3680 lncRNAs were picked out to reconstruct the co-expression networks using the RTN package. We used a total of 1000 permutations to predict these networks, and this analysis was performed considering three levels of significance, *p* < 10^−6^, *p* < 10^−7^, and *p* < 10^−8^ for better visualization of the most significant co-expression networks. To ensure a higher level of stringency of the analysis, we used the co-expression networks computed at the significance level of *p* < 10^−8^. 

The regulon’s activity heatmap was also performed using RTN package. Ranked differential Enrichment Score (dES) plot for the BRCA cohort and status of key attributes plot were constructed using RTN Survival package [41].

### 4.3. Regulon Filtering in Breast Cancer Molecular Subtypes

After the reconstruction of 3,680 co-expression networks, we exclude those that exhibit size less than 15 targets and those who had no Differential Enrichment Score (dES) −0.5 < dES > 0.5, thus, avoiding the selection of networks with small informative value. This selection resulted in a final number of 84 co-expression networks.

These 84 co-expression networks were then stratified according to breast cancer molecular subtype: Basal-like, HER2-enriched, Luminal A, and Luminal B. Normal-like samples were withdrawn from the analysis because of the small sample number (*n* = 7). The non-parametric Kruskal-Wallis test and post hoc Dunn’s test were used to verify the difference in the variance of dES comparing the breast cancer subtypes for the 84 regulatory networks selected. For additional analysis, only networks with *p*-value < 0.05 in both tests were considered.

At this point, we focus on the subsequent analyzes on the co-expression networks with the greatest difference of dES value among the subtypes of greatest differences in prognosis, luminal A and Basal-like. With this criterion, we aim to select co-expression networks that are differentially regulated between these subtypes. This selection resulted in 40 co-expression networks evaluated by available literature data, regarding the lncRNAs used to construct the co-expression networks and by the identification of genes previously described as relevant in mammary carcinogenesis.

### 4.4. Cancer-Related Processes Enrichment Analysis

The enrichment analysis of the co-expression networks was performed using Molecular Signatures Database (MSigDB). Within this database, we use the genetic signatures contained in the collection called “Hallmarks”. This group are coherently expressed signatures derived by aggregating many MSigDB gene sets to represent well-defined biological states or processes. In total, 50 genetic signatures of the “Hallmarks” collection are deposited and these are available at https://www.gsea-msigdb.org/gsea/msigdb/genesets.jsp?collection=H (accessed on 1 January 2021). The graphical representation of the enriched hallmarks was made using the ggplot2 package.

### 4.5. Breast Cancer Tissue Sample Characterization

A total of 55 breast tissue samples from different patients, predominantly of European descent, who had been diagnosed with breast cancer were collected during primary surgery at Hospital Nossa Senhora Das Graças (HNSG) from Curitiba. Clinical and histopathologic data of the patients were collected directly from the medical records in a coded manner without patient identifiers. The samples were classified according to their immunohistochemical profile according to Goldhirsch et al. [42]. In total, 30 samples classified as Luminal A and 25 as Triple negative were used. This study was approved by the Brazilian Commission of Ethics in Research (CONEP) under the number CAAE 67400917.3.0000.55 following Brazilian Federal laws in 20/02/2003. All participants signed informed written consent following the principles of the Declaration of Helsinki.

### 4.6. RNA Isolation and Expression Quantification

RNA isolation of the fresh tumor samples and cell lines was performed using RecoverAll Total Nucleic Acid Isolation Kit (Invitrogen, Carlsbad, CA, USA) and TRIzol^®^ Reagent (Invitrogen, Carlsbad, CA, USA) respectively, according to the manufacturer’s protocol. After the isolation, 1000 ng of RNA (each sample) were treated with DNAse I (Invitrogen, Carlsbad, CA, USA) and converted into cDNA using the SuperScript III enzyme (Invitrogen, Carlsbad, CA, USA) using random hemaxers, and following the manufacturer’s protocol. Real-time PCR was performed using the Viia-7 Sequence Detection Systems (Applied Biosystems, San Francisco, CA, USA) with Power SYBR Green PCR Master Mix (Applied Biosystems). For each reaction, we used RT- as a control and as a normalizer between the PCR reactions a cDNA pool of breast cancer tumor lines. The gene expression was determined using the 2^−ΔΔCt^ method [43], using β-glucuronidase (*GUS*β) and actin-beta (*ACTB*) expression as normalizers. The reactions were performed in triplicate, and negative control was included for each set of primers in each batch. The primer sequences are organized in Supplementary Table S1.

### 4.7. Cell Culture and Growth Conditions

MCF-7 and ZR-75-1cells were cultured in RPMI medium (Gibco, Grand Island, NY, USA) supplemented with 10% FBS (Gibco) and 1% Penicillin-Streptomycin and maintained a humidified incubator with 5% CO_2_.

### 4.8. siRNA Treatment

A total of 6 × 10^5^ MCF-7 and ZR75-1 cells were seeded onto six-well plates, reaching a total of 50–70% confluence. The cells were transfected with 125 nM siRNA scramble and siRNA LINC00504 (from the catalog of the company ThermoFisher, Waltham, MA, USA), number 4392420). Cells were reverse transfected using Lipofectamine 2000 reagent (Invitrogen), according to the manufacturer’s protocol and evaluated after 24, 48, and 72 h.

### 4.9. Cell Viability

For cell viability assay, MCF-7 and ZR75-1 cells treated with siRNA scramble and siLINC00504 were plated in 96 well plates. A total of 500 cells were seeded in each well. The cell viability was evaluated after 48, 72, and 96 h of transfection. On each day, the media were replaced with 100 µL of fresh media + 10 µL Resazurin dye (7-hydroxy-3H-phenoxazin-3-one 10-oxide) at concentration of 0.1 mg/mL and incubated for 4 h, and read at 600 ηm and 570 ηm using TECAN Infinite^®^ 200 PRO system. The experiment was made in triplicate.

### 4.10. Colony-Forming Assay

The cells were seeded in a 6-well plate (1000 cells each well) and grew for 25 days at 37 °C in a 5% CO_2_ humidified incubator after treatment with siLINC00504 or siRNA scramble. The cells were fixed with 100% methanol for 20 min, stained with 1% crystal violet for 5 min, and washed with water until excess dye is removed (every step was performed at room temperature). The experiment was carried out in triplicate.

### 4.11. Apoptosis

The apoptosis ratio was analyzed using the Annexin V-FITC Apoptosis Detection Kit. Forty-eight hours after transfection, MCF-7 and ZR75-1 cells were harvested and resuspended in a binding buffer containing Annexin V-FITC and P.I. (propidium iodide) according to its instructions. The samples were analyzed by flow cytometry (B.D. Biosciences, San Jose, CA, USA). Cells were discriminated into viable cells, necrotic cells, early apoptosis, and later apoptosis cells using the BD FACSVantage™ cytofluorimeter (B.D. Biosciences, USA), and then the percentages of apoptotic cells from each group were compared. Data analysis was performed using FlowJo software (v.10). The experiment was performed in triplicate.

### 4.12. Statistical Analysis

Data are presented as mean ± S.D. Statistical analysis of the data was performed by Student’s *t*-test using R (version 3.6.3) (http:///www.r-project.org/ (accessed on 1 January 2021)). *p*-values of ≤0.05 were considered statistically significant.

## Figures and Tables

**Table 1:** LINC00504 co-expression network elements detailing.

Network Element	Mutual Information	logFC
*CPEB2*	0.40	1.85
CPEB2-DT	−0.11	−0.05
*ESR1*	0.23	6.96
*FOXA1*	0.20	6.21
*GATA3*	0.24	4.33

logFC—based on the comparison luminal A x basal-like (*p* < 0.05).

## Data Availability

Data is contained within the article or Supplementary Materials.

## Supplementary Materials

The following are available online at https://www.mdpi.com/1422-0067/22/5/2420/s1. Table S1: Primers sequences utilized in RT-qPCR; Table S2: 84 LncRNAs co-expression networks after dES filtering; Table S3: 40 LncRNAs co-expression networks after luminal A and basal-like extratification filtering; Table S4: Common mRNAs identified in CPEB2 coimunopreciptation and LINC00504 co-expression network; Figure S1: LINC00504 relative expression in the tested breast cancer cell lines; Figure S2: Representative figure of colony formation assay in MCF-7 and ZR75-1 cell lines.
