# Supplementary material for: Novel lncRNAs Co-Expression Networks Identifies LINC00504 with Oncogenic Role in Luminal A Breast Cancer Cells
Source: Int J Mol Sci. 2021 Feb 28;22(5):2420. doi: 10.3390/ijms22052420 (PMC7957645; doi:10.3390/ijms22052420)
Supplement: Supplementary file 1 [file ijms-22-02420-s001.zip › Sup Material.zip!/Supplementary Table 1.docx]

| **Gene** | **Primer sequence** |
| --- | --- |
| LINC00504 | **F**: GAGCTGAGGCAGACGATGTAA  **R**: ACCCACTTCACTTCACCCAAG |
| *ACTB* | **F**: CCGATCCACACGGAGTACTTG  **R**: GGCACCCAGCACAATGAAG |
| *GUS-β* | **F**: CCGAGTGAAGATCCCCTTTTTA  **R**: GAAAATATGTGGTTGGAGAGCTCATT |
